# Undue Rigor in the Management of the Sick

**Published:** 1863-10

**Authors:** 


### UNDUE RIGOR IN THE MANAGEMENT OF THE SICK.

Next to the professional tyranny of overdosing, we would set that which seems to take delight in placing the unfortunate patient under excessive restraint in matters of diet and regimen. There is a class of physicians, happily growing smaller every day, who seem to think that the moment that a man gets sick all his natural tastes and appetites must be put in the closest bondage. Sickness being an abnormal condition, of course the normal demands of the system must be opposed, to bring the whole operation of the machine into consistent working. The food must be entirely different from that which is palatable at other times, and the ingenuity of friends and attendants must be taxed to provide substitutes for the usual beverages of health. We think we are speaking to the experience of every physician when we say that very often these well-meant efforts are a source of infinite disgust and discomfort to the patient. Some physicians there are who are so constituted that the bare suggestion by the patient of some article of food or medicine is enough to set them in dead opposition to it. Nothing can produce a stronger dislike on the part of an intelligent patient than such a display of professional authority. Well do we remember the vehement expressions of a sufferer from such severity of discipline, when describing a series of such "slips between the cup and the lip" by which he had been made to feel the authority of his medical adviser while recovering from a severe surgical operation. He was fully possessed with the idea that the interdict was merely an exercise of arbitrary power, and his experience in one instance, when the prohibition came too late, only confirmed this impression.

Cold water is an innocent fluid—of late years it has had its extravagant admirers even—and yet how common it is for patients to be unduly restrained in the use of it. How many unfortunate infants, gasping for it in the parched and fervid heat of scarlet fever, are compelled to swallow saffron tea instead! In such cases we know the fear of nature's refreshing draught is much more apt to be on the side of the parents and friends than the physician. And yet this is not always so.—Not long since we saw a patient, an adult, suffering from severe pneumonia. Some dissatisfaction on the part of the patient led her to call in another practitioner. "Doctor," said she, "is there any objection to my drinking cold water?"—"Certainly not," was the answer. "Why," said the patient, with a look of the greatest satisfaction and relief, "Dr. —— wouldn't let me touch a drop of it." The nurse then joined the colloquy, and confessed that she had "*unbeknownst*" to the doctor, given it to her, but it was under a fearful sense of responsibility.

In conclusion, then, let us bear in mind that *natura duce* should be our motto in matters of diet and regimen with the sick, as it is in all other matters of treatment. To most of our readers we know such a suggestion is superfluous, and almost demanding apology. That thirsty sufferer, then, must be our excuse; we trust it is a sufficient one.—*Boston Med. Jour.*

